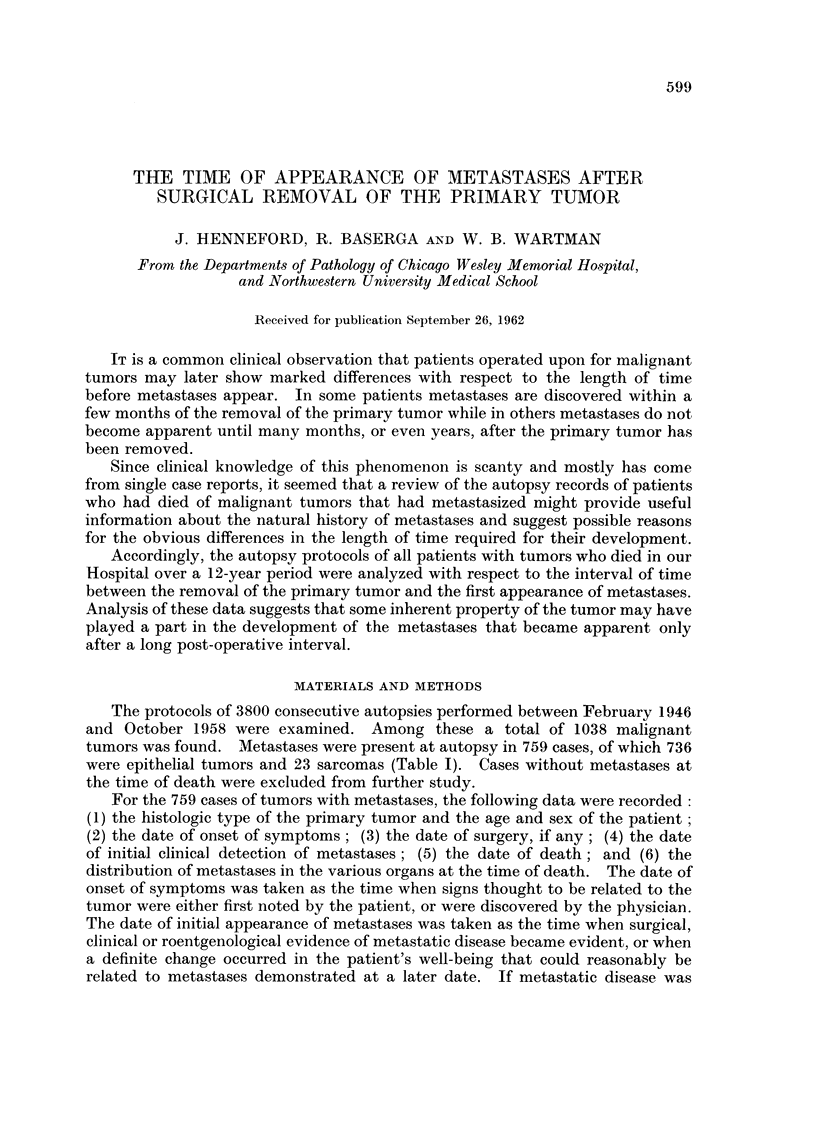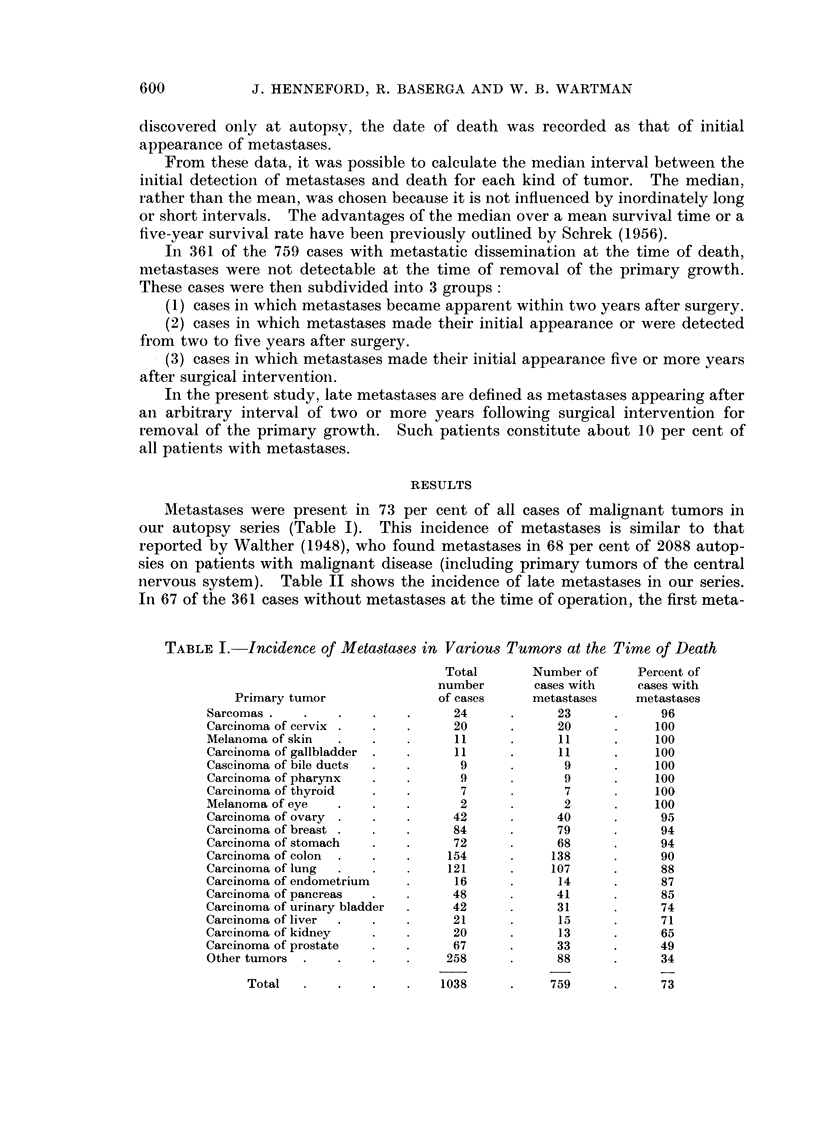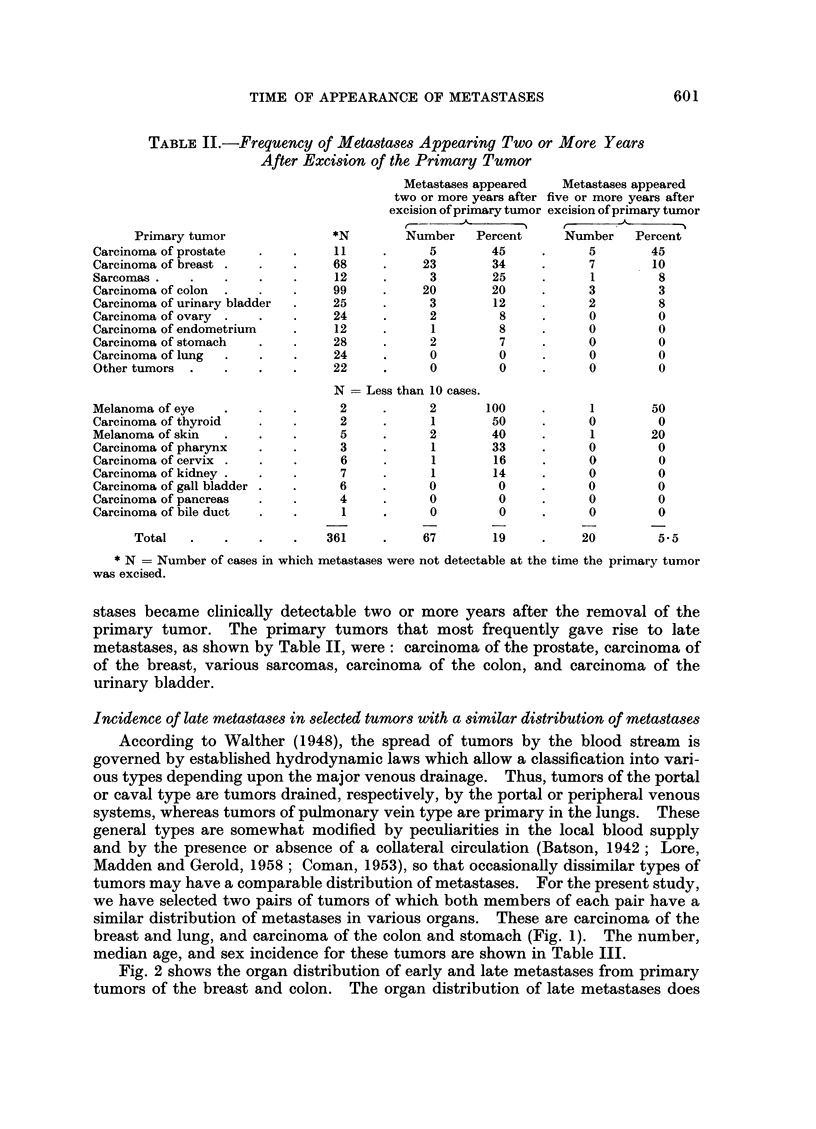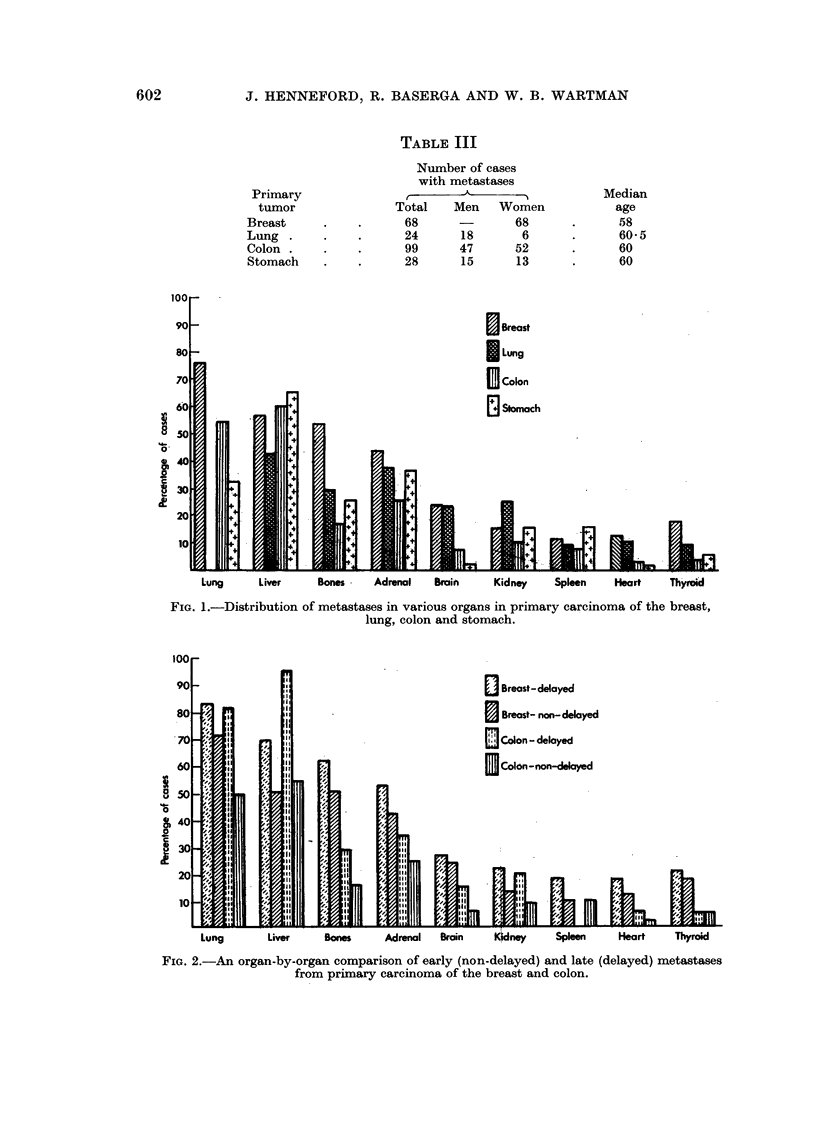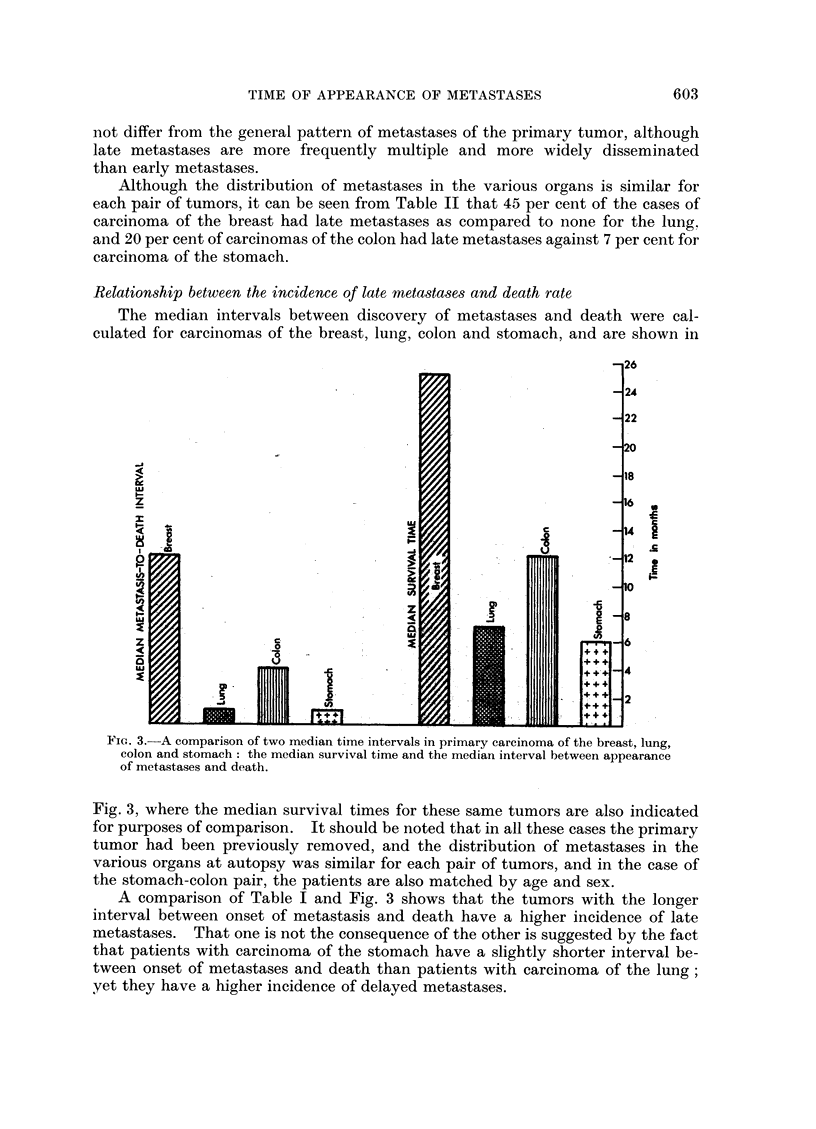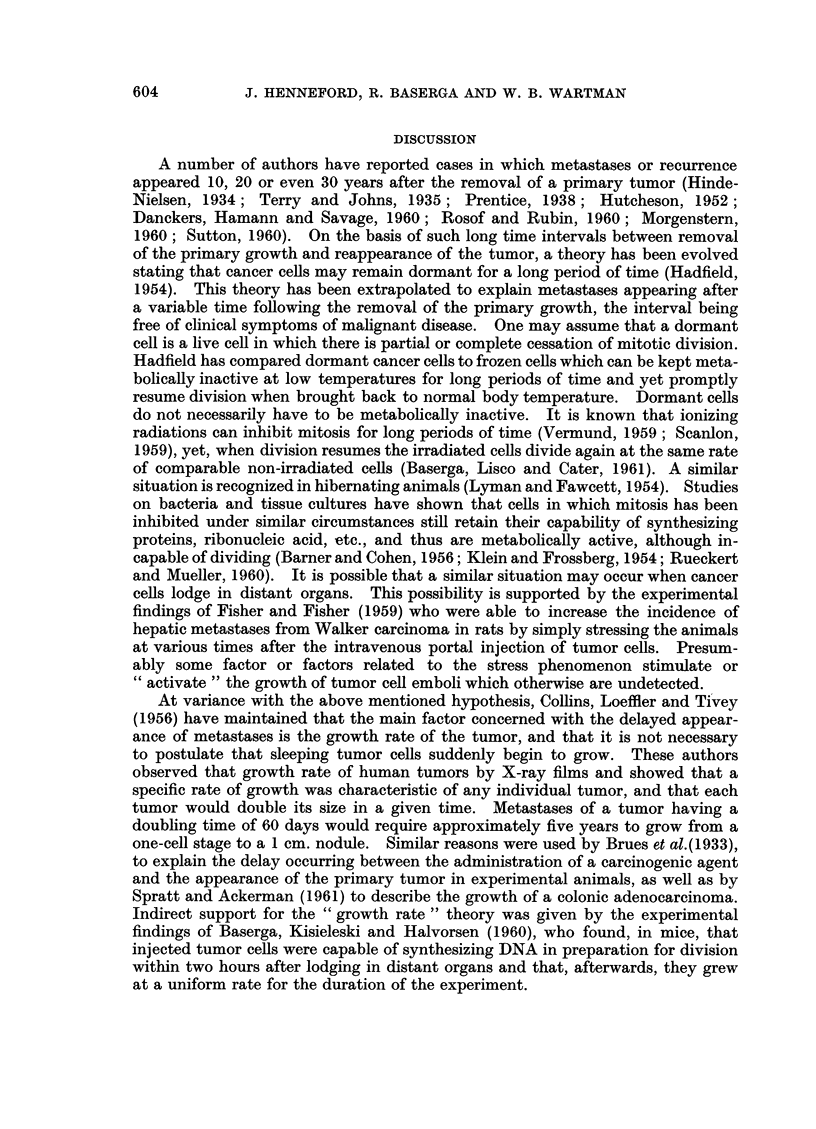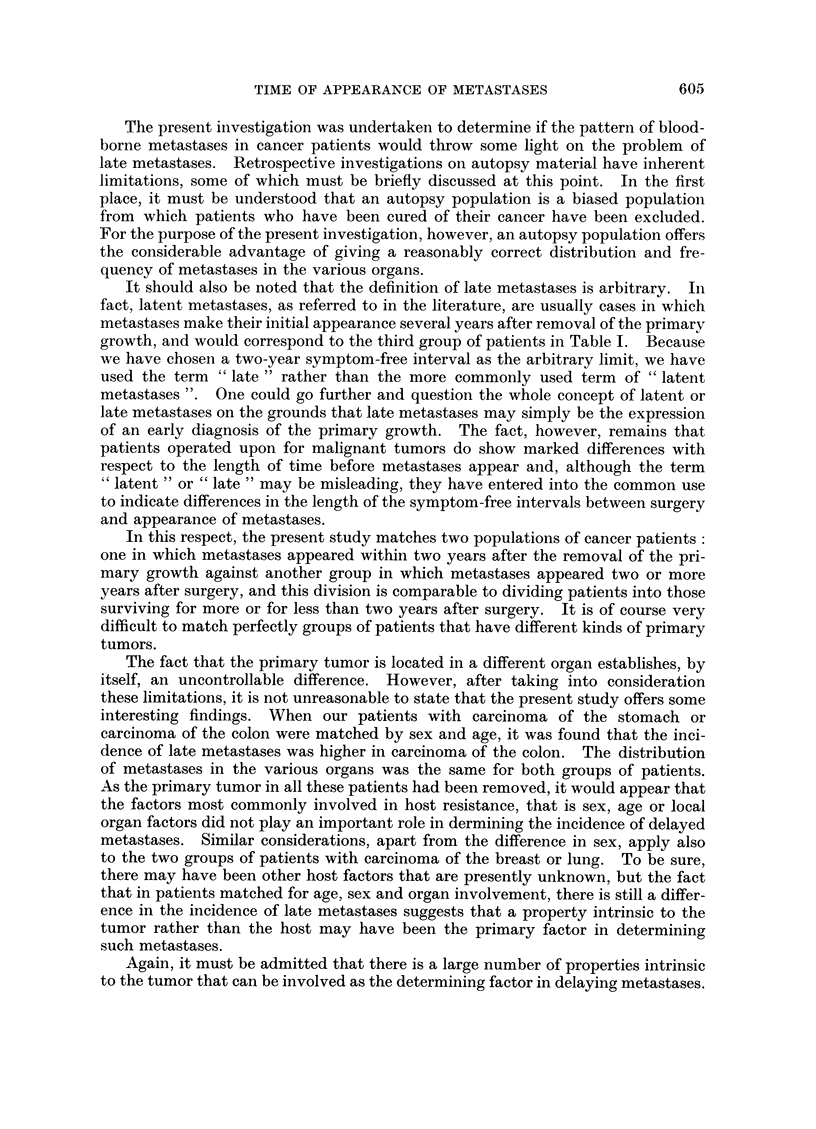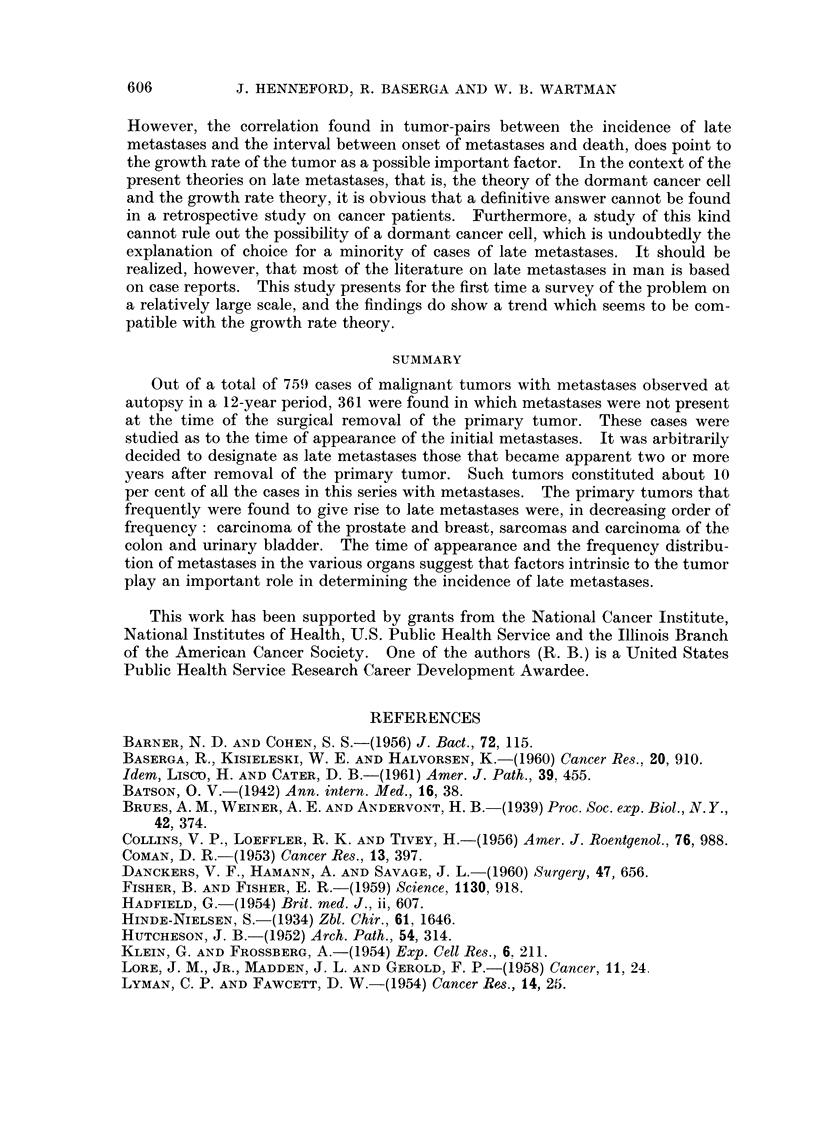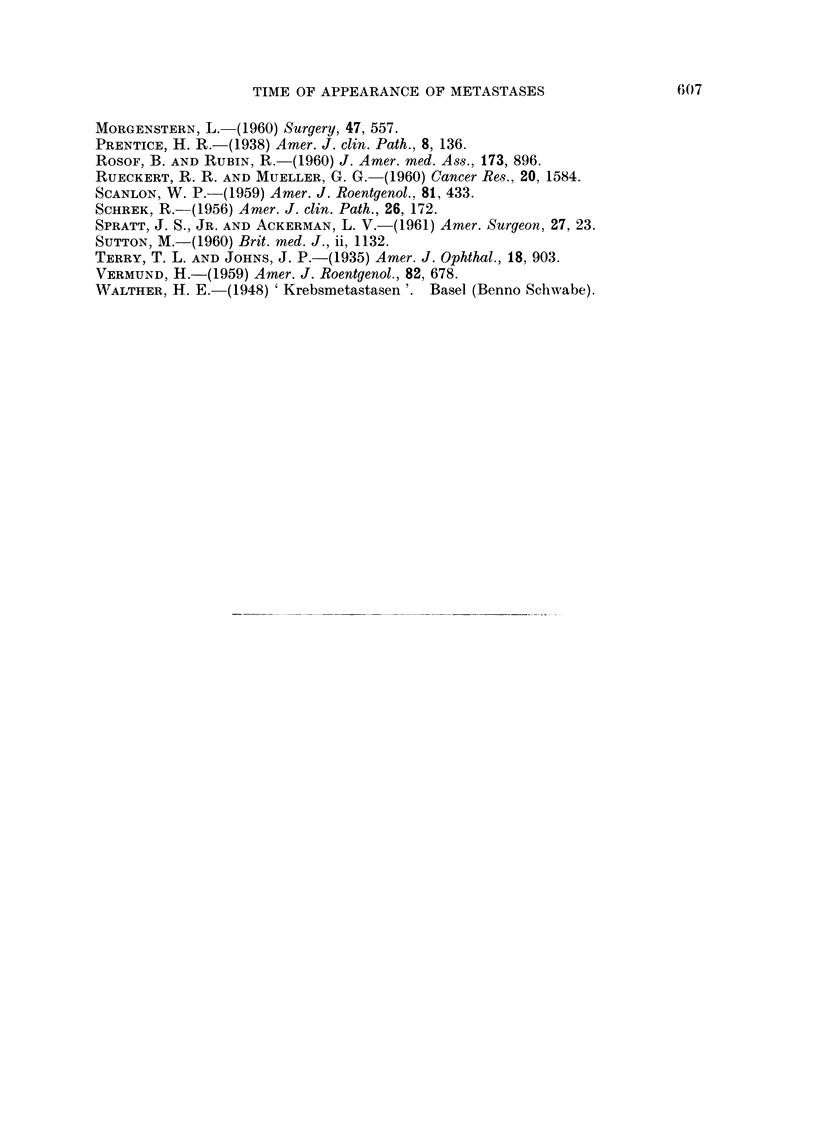# The Time of Appearance of Metastases After Surgical Removal of the Primary Tumor

**DOI:** 10.1038/bjc.1962.70

**Published:** 1962-12

**Authors:** J. Henneford, R. Baserga, W. B. Wartman


### THE TIME OF APPEARANCE OF METASTASES AFTER SURGICAL REMOVAL OF THE PRIMARY TUMOR

J. HENNEFORD, R. BASERGA AND W. B. WARTMAN

*From the Departments of Pathology of Chicago Wesley Memorial Hospital, and Northwestern University Medical School*

Received for publication September 26, 1962

It is a common clinical observation that patients operated upon for malignant tumors may later show marked differences with respect to the length of time before metastases appear. In some patients metastases are discovered within a few months of the removal of the primary tumor while in others metastases do not become apparent until many months, or even years, after the primary tumor has been removed.

Since clinical knowledge of this phenomenon is scanty and mostly has come from single case reports, it seemed that a review of the autopsy records of patients who had died of malignant tumors that had metastasized might provide useful information about the natural history of metastases and suggest possible reasons for the obvious differences in the length of time required for their development.

Accordingly, the autopsy protocols of all patients with tumors who died in our Hospital over a 12-year period were analyzed with respect to the interval of time between the removal of the primary tumor and the first appearance of metastases. Analysis of these data suggests that some inherent property of the tumor may have played a part in the development of the metastases that became apparent only after a long post-operative interval.

### MATERIALS AND METHODS

The protocols of 3800 consecutive autopsies performed between February 1946 and October 1958 were examined. Among these a total of 1038 malignant tumors was found. Metastases were present at autopsy in 759 cases, of which 736 were epithelial tumors and 23 sarcomas (Table I). Cases without metastases at the time of death were excluded from further study.

For the 759 cases of tumors with metastases, the following data were recorded : (1) the histologic type of the primary tumor and the age and sex of the patient ; (2) the date of onset of symptoms ; (3) the date of surgery, if any ; (4) the date of initial clinical detection of metastases ; (5) the date of death ; and (6) the distribution of metastases in the various organs at the time of death. The date of onset of symptoms was taken as the time when signs thought to be related to the tumor were either first noted by the patient, or were discovered by the physician. The date of initial appearance of metastases was taken as the time when surgical, clinical or roentgenological evidence of metastatic disease became evident, or when a definite change occurred in the patient's well-being that could reasonably be related to metastases demonstrated at a later date. If metastatic disease was discovered only at autopsy, the date of death was recorded as that of initial appearance of metastases.

From these data, it was possible to calculate the median interval between the initial detection of metastases and death for each kind of tumor. The median, rather than the mean, was chosen because it is not influenced by inordinately long or short intervals. The advantages of the median over a mean survival time or a five-year survival rate have been previously outlined by Schrek (1956).

In 361 of the 759 cases with metastatic dissemination at the time of death, metastases were not detectable at the time of removal of the primary growth. These cases were then subdivided into 3 groups :

(1) cases in which metastases became apparent within two years after surgery.

(2) cases in which metastases made their initial appearance or were detected from two to five years after surgery.

(3) cases in which metastases made their initial appearance five or more years after surgical intervention.

In the present study, late metastases are defined as metastases appearing after an arbitrary interval of two or more years following surgical intervention for removal of the primary growth. Such patients constitute about 10 per cent of all patients with metastases.

### RESULTS

Metastases were present in 73 per cent of all cases of malignant tumors in our autopsy series (Table I). This incidence of metastases is similar to that reported by Walther (1948), who found metastases in 68 per cent of 2088 autopsies on patients with malignant disease (including primary tumors of the central nervous system). Table II shows the incidence of late metastases in our series. In 67 of the 361 cases without metastases at the time of operation, the first meta-

TABLE I.—*Incidence of Metastases in Various Tumors at the Time of Death*

| Primary tumor | Total number of cases | Number of cases with metastases | Percent of cases with metastases |
|---|---|---|---|
| Sarcomas | 24 | 23 | 96 |
| Carcinoma of cervix | 20 | 20 | 100 |
| Melanoma of skin | 11 | 11 | 100 |
| Carcinoma of gallbladder | 11 | 11 | 100 |
| Cascinoma of bile ducts | 9 | 9 | 100 |
| Carcinoma of pharynx | 9 | 9 | 100 |
| Carcinoma of thyroid | 7 | 7 | 100 |
| Melanoma of eye | 2 | 2 | 100 |
| Carcinoma of ovary | 42 | 40 | 95 |
| Carcinoma of breast | 84 | 79 | 94 |
| Carcinoma of stomach | 72 | 68 | 94 |
| Carcinoma of colon | 154 | 138 | 90 |
| Carcinoma of lung | 121 | 107 | 88 |
| Carcinoma of endometrium | 16 | 14 | 87 |
| Carcinoma of pancreas | 48 | 41 | 85 |
| Carcinoma of urinary bladder | 42 | 31 | 74 |
| Carcinoma of liver | 21 | 15 | 71 |
| Carcinoma of kidney | 20 | 13 | 65 |
| Carcinoma of prostate | 67 | 33 | 49 |
| Other tumors | 258 | 88 | 34 |
| Total | 1038 | 759 | 73 |

TABLE II.—*Frequency of Metastases Appearing Two or More Years After Excision of the Primary Tumor*

| Primary tumor | *N | Metastases appeared two or more years after excision of primary tumor | | Metastases appeared five or more years after excision of primary tumor | |
|---|---|---|---|---|---|
| | | Number | Percent | Number | Percent |
| Carcinoma of prostate | 11 | 5 | 45 | 5 | 45 |
| Carcinoma of breast | 68 | 23 | 34 | 7 | 10 |
| Sarcomas | 12 | 3 | 25 | 1 | 8 |
| Carcinoma of colon | 99 | 20 | 20 | 3 | 3 |
| Carcinoma of urinary bladder | 25 | 3 | 12 | 2 | 8 |
| Carcinoma of ovary | 24 | 2 | 8 | 0 | 0 |
| Carcinoma of endometrium | 12 | 1 | 8 | 0 | 0 |
| Carcinoma of stomach | 28 | 2 | 7 | 0 | 0 |
| Carcinoma of lung | 24 | 0 | 0 | 0 | 0 |
| Other tumors | 22 | 0 | 0 | 0 | 0 |
| N = Less than 10 cases. | | | | | |
| Melanoma of eye | 2 | 2 | 100 | 1 | 50 |
| Carcinoma of thyroid | 2 | 1 | 50 | 0 | 0 |
| Melanoma of skin | 5 | 2 | 40 | 1 | 20 |
| Carcinoma of pharynx | 3 | 1 | 33 | 0 | 0 |
| Carcinoma of cervix | 6 | 1 | 16 | 0 | 0 |
| Carcinoma of kidney | 7 | 1 | 14 | 0 | 0 |
| Carcinoma of gall bladder | 6 | 0 | 0 | 0 | 0 |
| Carcinoma of pancreas | 4 | 0 | 0 | 0 | 0 |
| Carcinoma of bile duct | 1 | 0 | 0 | 0 | 0 |
| Total | 361 | 67 | 19 | 20 | 5·5 |

* N = Number of cases in which metastases were not detectable at the time the primary tumor was excised.

stases became clinically detectable two or more years after the removal of the primary tumor. The primary tumors that most frequently gave rise to late metastases, as shown by Table II, were : carcinoma of the prostate, carcinoma of of the breast, various sarcomas, carcinoma of the colon, and carcinoma of the urinary bladder.

*Incidence of late metastases in selected tumors with a similar distribution of metastases*

According to Walther (1948), the spread of tumors by the blood stream is governed by established hydrodynamic laws which allow a classification into various types depending upon the major venous drainage. Thus, tumors of the portal or caval type are tumors drained, respectively, by the portal or peripheral venous systems, whereas tumors of pulmonary vein type are primary in the lungs. These general types are somewhat modified by peculiarities in the local blood supply and by the presence or absence of a collateral circulation (Batson, 1942 ; Lore, Madden and Gerold, 1958 ; Coman, 1953), so that occasionally dissimilar types of tumors may have a comparable distribution of metastases. For the present study, we have selected two pairs of tumors of which both members of each pair have a similar distribution of metastases in various organs. These are carcinoma of the breast and lung, and carcinoma of the colon and stomach (Fig. 1). The number, median age, and sex incidence for these tumors are shown in Table III.

Fig. 2 shows the organ distribution of early and late metastases from primary tumors of the breast and colon. The organ distribution of late metastases does

TABLE III

| Primary tumor | Number of cases with metastases: Total | Men | Women | Median age |
|---|---|---|---|---|
| Breast | 68 | — | 68 | 58 |
| Lung | 24 | 18 | 6 | 60·5 |
| Colon | 99 | 47 | 52 | 60 |
| Stomach | 28 | 15 | 13 | 60 |

FIG. 1.—Distribution of metastases in various organs in primary carcinoma of the breast, lung, colon and stomach.

FIG. 2.—An organ-by-organ comparison of early (non-delayed) and late (delayed) metastases from primary carcinoma of the breast and colon.

not differ from the general pattern of metastases of the primary tumor, although late metastases are more frequently multiple and more widely disseminated than early metastases.

Although the distribution of metastases in the various organs is similar for each pair of tumors, it can be seen from Table II that 45 per cent of the cases of carcinoma of the breast had late metastases as compared to none for the lung, and 20 per cent of carcinomas of the colon had late metastases against 7 per cent for carcinoma of the stomach.

*Relationship between the incidence of late metastases and death rate*

The median intervals between discovery of metastases and death were calculated for carcinomas of the breast, lung, colon and stomach, and are shown in

Fig. 3.—A comparison of two median time intervals in primary carcinoma of the breast, lung, colon and stomach : the median survival time and the median interval between appearance of metastases and death.

Fig. 3, where the median survival times for these same tumors are also indicated for purposes of comparison. It should be noted that in all these cases the primary tumor had been previously removed, and the distribution of metastases in the various organs at autopsy was similar for each pair of tumors, and in the case of the stomach-colon pair, the patients are also matched by age and sex.

A comparison of Table I and Fig. 3 shows that the tumors with the longer interval between onset of metastasis and death have a higher incidence of late metastases. That one is not the consequence of the other is suggested by the fact that patients with carcinoma of the stomach have a slightly shorter interval between onset of metastases and death than patients with carcinoma of the lung ; yet they have a higher incidence of delayed metastases.

### DISCUSSION

A number of authors have reported cases in which metastases or recurrence appeared 10, 20 or even 30 years after the removal of a primary tumor (Hinde-Nielsen, 1934; Terry and Johns, 1935; Prentice, 1938; Hutcheson, 1952; Danckers, Hamann and Savage, 1960; Rosof and Rubin, 1960; Morgenstern, 1960; Sutton, 1960). On the basis of such long time intervals between removal of the primary growth and reappearance of the tumor, a theory has been evolved stating that cancer cells may remain dormant for a long period of time (Hadfield, 1954). This theory has been extrapolated to explain metastases appearing after a variable time following the removal of the primary growth, the interval being free of clinical symptoms of malignant disease. One may assume that a dormant cell is a live cell in which there is partial or complete cessation of mitotic division. Hadfield has compared dormant cancer cells to frozen cells which can be kept metabolically inactive at low temperatures for long periods of time and yet promptly resume division when brought back to normal body temperature. Dormant cells do not necessarily have to be metabolically inactive. It is known that ionizing radiations can inhibit mitosis for long periods of time (Vermund, 1959; Scanlon, 1959), yet, when division resumes the irradiated cells divide again at the same rate of comparable non-irradiated cells (Baserga, Lisco and Cater, 1961). A similar situation is recognized in hibernating animals (Lyman and Fawcett, 1954). Studies on bacteria and tissue cultures have shown that cells in which mitosis has been inhibited under similar circumstances still retain their capability of synthesizing proteins, ribonucleic acid, etc., and thus are metabolically active, although incapable of dividing (Barner and Cohen, 1956; Klein and Frossberg, 1954; Rueckert and Mueller, 1960). It is possible that a similar situation may occur when cancer cells lodge in distant organs. This possibility is supported by the experimental findings of Fisher and Fisher (1959) who were able to increase the incidence of hepatic metastases from Walker carcinoma in rats by simply stressing the animals at various times after the intravenous portal injection of tumor cells. Presumably some factor or factors related to the stress phenomenon stimulate or "activate" the growth of tumor cell emboli which otherwise are undetected.

At variance with the above mentioned hypothesis, Collins, Loeffler and Tivey (1956) have maintained that the main factor concerned with the delayed appearance of metastases is the growth rate of the tumor, and that it is not necessary to postulate that sleeping tumor cells suddenly begin to grow. These authors observed that growth rate of human tumors by X-ray films and showed that a specific rate of growth was characteristic of any individual tumor, and that each tumor would double its size in a given time. Metastases of a tumor having a doubling time of 60 days would require approximately five years to grow from a one-cell stage to a 1 cm. nodule. Similar reasons were used by Brues *et al.*(1933), to explain the delay occurring between the administration of a carcinogenic agent and the appearance of the primary tumor in experimental animals, as well as by Spratt and Ackerman (1961) to describe the growth of a colonic adenocarcinoma. Indirect support for the "growth rate" theory was given by the experimental findings of Baserga, Kisieleski and Halvorsen (1960), who found, in mice, that injected tumor cells were capable of synthesizing DNA in preparation for division within two hours after lodging in distant organs and that, afterwards, they grew at a uniform rate for the duration of the experiment.

The present investigation was undertaken to determine if the pattern of blood-borne metastases in cancer patients would throw some light on the problem of late metastases. Retrospective investigations on autopsy material have inherent limitations, some of which must be briefly discussed at this point. In the first place, it must be understood that an autopsy population is a biased population from which patients who have been cured of their cancer have been excluded. For the purpose of the present investigation, however, an autopsy population offers the considerable advantage of giving a reasonably correct distribution and frequency of metastases in the various organs.

It should also be noted that the definition of late metastases is arbitrary. In fact, latent metastases, as referred to in the literature, are usually cases in which metastases make their initial appearance several years after removal of the primary growth, and would correspond to the third group of patients in Table I. Because we have chosen a two-year symptom-free interval as the arbitrary limit, we have used the term " late " rather than the more commonly used term of " latent metastases ". One could go further and question the whole concept of latent or late metastases on the grounds that late metastases may simply be the expression of an early diagnosis of the primary growth. The fact, however, remains that patients operated upon for malignant tumors do show marked differences with respect to the length of time before metastases appear and, although the term " latent " or " late " may be misleading, they have entered into the common use to indicate differences in the length of the symptom-free intervals between surgery and appearance of metastases.

In this respect, the present study matches two populations of cancer patients : one in which metastases appeared within two years after the removal of the primary growth against another group in which metastases appeared two or more years after surgery, and this division is comparable to dividing patients into those surviving for more or for less than two years after surgery. It is of course very difficult to match perfectly groups of patients that have different kinds of primary tumors.

The fact that the primary tumor is located in a different organ establishes, by itself, an uncontrollable difference. However, after taking into consideration these limitations, it is not unreasonable to state that the present study offers some interesting findings. When our patients with carcinoma of the stomach or carcinoma of the colon were matched by sex and age, it was found that the incidence of late metastases was higher in carcinoma of the colon. The distribution of metastases in the various organs was the same for both groups of patients. As the primary tumor in all these patients had been removed, it would appear that the factors most commonly involved in host resistance, that is sex, age or local organ factors did not play an important role in dermining the incidence of delayed metastases. Similar considerations, apart from the difference in sex, apply also to the two groups of patients with carcinoma of the breast or lung. To be sure, there may have been other host factors that are presently unknown, but the fact that in patients matched for age, sex and organ involvement, there is still a difference in the incidence of late metastases suggests that a property intrinsic to the tumor rather than the host may have been the primary factor in determining such metastases.

Again, it must be admitted that there is a large number of properties intrinsic to the tumor that can be involved as the determining factor in delaying metastases.

However, the correlation found in tumor-pairs between the incidence of late metastases and the interval between onset of metastases and death, does point to the growth rate of the tumor as a possible important factor. In the context of the present theories on late metastases, that is, the theory of the dormant cancer cell and the growth rate theory, it is obvious that a definitive answer cannot be found in a retrospective study on cancer patients. Furthermore, a study of this kind cannot rule out the possibility of a dormant cancer cell, which is undoubtedly the explanation of choice for a minority of cases of late metastases. It should be realized, however, that most of the literature on late metastases in man is based on case reports. This study presents for the first time a survey of the problem on a relatively large scale, and the findings do show a trend which seems to be compatible with the growth rate theory.

### SUMMARY

Out of a total of 759 cases of malignant tumors with metastases observed at autopsy in a 12-year period, 361 were found in which metastases were not present at the time of the surgical removal of the primary tumor. These cases were studied as to the time of appearance of the initial metastases. It was arbitrarily decided to designate as late metastases those that became apparent two or more years after removal of the primary tumor. Such tumors constituted about 10 per cent of all the cases in this series with metastases. The primary tumors that frequently were found to give rise to late metastases were, in decreasing order of frequency : carcinoma of the prostate and breast, sarcomas and carcinoma of the colon and urinary bladder. The time of appearance and the frequency distribution of metastases in the various organs suggest that factors intrinsic to the tumor play an important role in determining the incidence of late metastases.

This work has been supported by grants from the National Cancer Institute, National Institutes of Health, U.S. Public Health Service and the Illinois Branch of the American Cancer Society. One of the authors (R. B.) is a United States Public Health Service Research Career Development Awardee.

### REFERENCES

Barner, N. D. and Cohen, S. S.—(1956) *J. Bact.*, **72**, 115.
Baserga, R., Kisieleski, W. E. and Halvorsen, K.—(1960) *Cancer Res.*, **20**, 910.
*Idem*, Lisco, H. and Cater, D. B.—(1961) *Amer. J. Path.*, **39**, 455.
Batson, O. V.—(1942) *Ann. intern. Med.*, **16**, 38.
Brues, A. M., Weiner, A. E. and Andervont, H. B.—(1939) *Proc. Soc. exp. Biol., N.Y.*, **42**, 374.
Collins, V. P., Loeffler, R. K. and Tivey, H.—(1956) *Amer. J. Roentgenol.*, **76**, 988.
Coman, D. R.—(1953) *Cancer Res.*, **13**, 397.
Danckers, V. F., Hamann, A. and Savage, J. L.—(1960) *Surgery*, **47**, 656.
Fisher, B. and Fisher, E. R.—(1959) *Science*, **1130**, 918.
Hadfield, G.—(1954) *Brit. med. J.*, ii, 607.
Hinde-Nielsen, S.—(1934) *Zbl. Chir.*, **61**, 1646.
Hutcheson, J. B.—(1952) *Arch. Path.*, **54**, 314.
Klein, G. and Frossberg, A.—(1954) *Exp. Cell Res.*, **6**, 211.
Lore, J. M., Jr., Madden, J. L. and Gerold, F. P.—(1958) *Cancer*, **11**, 24.
Lyman, C. P. and Fawcett, D. W.—(1954) *Cancer Res.*, **14**, 25.

MORGENSTERN, L.—(1960) *Surgery*, **47**, 557.
PRENTICE, H. R.—(1938) *Amer. J. clin. Path.*, **8**, 136.
ROSOF, B. AND RUBIN, R.—(1960) *J. Amer. med. Ass.*, **173**, 896.
RUECKERT, R. R. AND MUELLER, G. G.—(1960) *Cancer Res.*, **20**, 1584.
SCANLON, W. P.—(1959) *Amer. J. Roentgenol.*, **81**, 433.
SCHREK, R.—(1956) *Amer. J. clin. Path.*, **26**, 172.
SPRATT, J. S., JR. AND ACKERMAN, L. V.—(1961) *Amer. Surgeon*, **27**, 23.
SUTTON, M.—(1960) *Brit. med. J.*, ii, 1132.
TERRY, T. L. AND JOHNS, J. P.—(1935) *Amer. J. Ophthal.*, **18**, 903.
VERMUND, H.—(1959) *Amer. J. Roentgenol.*, **82**, 678.
WALTHER, H. E.—(1948) ' Krebsmetastasen '. Basel (Benno Schwabe).